# Do college entrance examination admission characteristics influence students’ college satisfaction? Evidence from China

**DOI:** 10.3389/fpsyg.2023.1115867

**Published:** 2023-03-22

**Authors:** Yifan Huang, Miao Huang, Huijuan Wang, Zhaojun Chen, Xinqiao Liu

**Affiliations:** ^1^Center for Higher Education Development Research/Institute of Education, Xiamen University, Xiamen, China; ^2^College of Humanities, Yantai Nanshan University, Yantai, China; ^3^Institute of Education, Xiamen University, Xiamen, China; ^4^School of Education, Tianjin University, Tianjin, China

**Keywords:** college entrance examination, first choice, admitted characteristics, propensity score matching, students’ college satisfaction

## Abstract

Students’ college satisfaction is an important part of measuring the quality of college teaching. The admission of college entrance exam is the first step for college students to enter colleges and corresponding majors. Whether they affect students’ college satisfaction after enrollment is related to the formulation and adjustment of college admission strategies and training methods. This paper is based on data from students in colleges in Beijing enrolled in the fall of 2006 and 2008 and adopts propensity score matching to analyze the influence of the admitted characteristics of college entrance exams, such as whether they were accepted by their first choice. We also further explored the heterogeneity. The empirical results show that whether the student was admitted to the first-choice college has a significant positive impact on overall satisfaction and academic and nonacademic satisfaction, while whether the student was admitted to the first-choice major has no significant impact on nonacademic satisfaction. In addition, making an independent major choice has a positive effect on the improvement of overall satisfaction and academic satisfaction, and the impact on overall satisfaction is even greater than that of being admitted to the first-choice major. The impact of the admission characteristics of college entrance examinations on the satisfaction of students in liberal arts and science and students of different types of colleges and universities presents different characteristics.

## 1. Introduction

As the main form of college admission and student enrollment in China, as well as an important channel for talent screening and social mobility, college entrance examinations are not only directly related to the applied school level, major type and future development of students but also have extraordinary significance for individuals, families, universities and even the whole higher education system.

The college admission policy is closely related to the application and admission mechanism, both of which will have a vital impact on students’ admission results and college satisfaction. However, most students still follow the rule of “score matching first, major selection second” due to a severe shortage of guidance in application and having no access to professional information before entering college (Zhang and Chen, 2015, 173–174 + 181). Moreover, students make their picks more based on the advice of parents and teachers rather than personal interests. A survey conducted by a third-party data company also shows that current majors for nearly a quarter of fresh students are not their preferred ones, and 29 percent decide to drop out because “their choices of majors do not match their expectations” (Chen, 2017).

In addition, with the continuous development of higher education marketization and the penetration of academic capitalism, the concept of service quality has gradually replaced the traditional concept of product quality and drawn researchers’ attention. Similarly, indicators such as functional quality (such as the quality of service process) and technical quality (such as the quality of talent training) are also incorporated in the quality assessment of higher education. In August 2012, the Ministry of Education issued a document requiring that the status quo of students’ learning satisfaction be included in the report of undergraduate teaching quality, and satisfaction has gradually become an important factor in evaluating college functions and the reference for college decision-making (Bao, 2014, 22–29 + 55).

Researchers in China have performed preliminary studies on students’ college satisfaction since the 1990s, but many findings have shown that students, especially fresh students, are not quite satisfied (Fan, 2011, 43–45 + 106). This will directly or indirectly increase the risk of students developing mental health problems such as anxiety and depression (Gao et al., 2020, 292–300; Liu X.C. et al., 2022, 860–873; Cao and Liu, 2022, 1,287–1,297; Liu A. et al., 2023, 1,442–1,457). At present, studies on factors affecting students’ college satisfaction mainly focus on the training process after admission, such as teaching quality and college environment, while little attention is given to the impact of the admitted characteristics of college entrance examinations. Therefore, the underlying reasons and influencing mechanisms of low satisfaction are exciting fields for exploration.

Based on the above analysis, the purpose of this paper is put forward: (1) Investigate the current situation of students’ college satisfaction; (2) Investigate the relationship between students’ admitted characteristics and students’ college satisfaction; (3) Analyze the difference in the influence of different groups of students’ admitted characteristics on students’ college satisfaction; and (4) Through the statistical analysis of the survey results, the conclusion is drawn, and suggestions are put forward for students’ voluntary choice of college entrance examination and universities’ improvement of college satisfaction.

## 2. Theoretical background and literature review

### 2.1. Theoretical background

The theory of relative deprivation, proposed by American sociologist S.A. Stouffer and developed by R.K. Merton indicates that it is easier for individuals to generate negative cognition and subjective experience by comparing with a given standard or reference object (Xiong and Ye, 2016, 438–453). Relative deprivation consists of two parts, namely, cognition (perceived that one’s expectations cannot be met) and affection (resulting in a sense of injustice, anger, and dissatisfaction; Bougie et al., 2011, 726–746). With their first choice unfulfilled and by comparing themselves with others after enrollment, students can easily generate a sense of relative deprivation, thereby reducing their satisfaction. In addition, self-decide theory (SDT) believes that individuals have the potential to make free choices after fully understanding their own needs and the surrounding environment, to stimulate internal motivation and to engage in exciting work (Zhang et al., 2010, 752–759). For students who can choose majors independently, intrinsic motivation for learning is more likely to be triggered, thereby positively affecting students’ college satisfaction.

### 2.2. Literature review

The concept of learning satisfaction has been the focus of study ever since the 1950s. Symonds (1955) explored the influence of learner satisfaction in the field of psychology and education. It is generally defined as a feeling or attitude of learners that their desires and needs can be fulfilled in learning activities or processes (Topala and Tomozii, 2014). Learning satisfaction, as the origin of higher education quality evaluation (Wen, 2015), is increasingly receiving attention from the academic community. Learning satisfaction is an indicator to measure whether learners achieve the expected learning outcomes (Martin, 1994). College students’ learning satisfaction is generally influenced at the individual and school levels. From the perspective of individual psychological factors, Liu X. et al. (2023) found that college students’ belief in a just world had a positive impact on their learning satisfaction. Chun-Hsiung Huang (2021) also found that dimensions of perceived usefulness, perceived ease of use, and learning motivation are the influencing factors of learning satisfaction. At the school level, learners’ satisfaction is affected by teaching mode, course content, and learning environment (Xu, 2018), and even the teaching quality (Adler et al., 2021). Teachers’ pre-service preparation affects student achievement and teaching quality (Liu X.Q. et al., 2023, 69). However, there is a lack of discussion of the factors before college students’ admission to college, and the key step in college entrance examination admission is selecting majors and voluntary reporting, which is likely to influence students’ learning satisfaction.

The literature on college admission mainly focuses on scores and reforms of application mechanisms (Nie, 2007, 23–26), while few studies reflect on college admission itself and subsequent training by comparing the performance of students with different admission characteristics after enrollment. Existing research can be broadly divided into three categories. Therefore, the first type explores the difference between different groups in filling out the college entrance examination. Students from different family backgrounds have different subject selection strategies and college admission opportunities due to differences in social class, culture, resources, and information (Wei et al., 2019, 39–48). In particular, the professional choices of students in rural areas are severely limited and lack freedom of choice and conditions (Qiuxiang et al., 2022, 51–58; Cao et al., 2023, 131), while city students are more willing to take risks than rural students (Qian, 2022, 29–34 + 4). On the other hand, not all students can choose the major they want. Under the strict restrictions of college major admission plans, students’ college entrance examination scores directly affect their eligibility for major selection. Students with score advantages have more room for major selection, while students with score disadvantages often have to accept major adjustments to ensure admission. Thus, when filling out the major application, there is a tendency to “make the most of it,” that is, some students view their college entrance examination scores as tools for major selection, not only choosing the subjects with the highest possibility of getting high scores (Chenghuo, 2018, 25–30) but also choosing the “hottest” or “best” major they can enter based on their scores, rather than their most interested major (Liao et al., 2017, 33–39 + 70).

The second type focuses on the performance of students with different admission routes and mainly studies two questions. First, the influence of the first-choice major or college on students’ academic prospects and professional interest. Relevant studies have shown that the admitted characteristics profoundly affect students’ subsequent development, and nonfirst-choice students (students whose colleges or majors are not their first choices) encounter extremely severe problems concerning academic adaptation, professional commitment, and mental health (Cai and Li, 2016, 66–74 + 2). One of the leading reasons is that students are dissatisfied with nonfirst-choice majors. Lower students’ college satisfaction will then influence their academic achievements (Cabrera et al., 1993, 123–139), physical and mental health (Liu, 2012, 22 + 53), etc., and improving students’ college satisfaction will promote and contribute to employment attitudes (Wang et al., 2013, 78–84), professional decision-making and prospects (Nauta, 2007, 446–462). However, the results show that its influence on students’ future development is gradually declining (Liu and Jiang, 2019, 22–25). For major-adjusted students, their professional interest is gradually increasing, although it is much lower than that of first-choice students in the freshman year (Liu and Jiang, 2018, 53–60). Second, there are subjective matching differences among students with varied admission procedures, such as unified examination and recommended admission. The findings show that admission procedures have a significant impact on the initial state and variation trend of individual subjective matching degree (Nie et al., 2014, 38–47).

The third type focuses on the correlation between the independent selection of majors and professional satisfaction. The findings show that independent selection can promote professional satisfaction by enhancing professional commitment (Ding, 2019, 27–33). Compared with making decisions before enrollment, choosing majors after admission benefits both academic interest (Ma et al., 2017, 131–144 + 190–191) and adaptation (Bartolj and Polanec, 2012, 996–1,016) and further improves students’ college satisfaction and enthusiasm. Moreover, students’ education investment can be more efficient and profitable (Malamud, 2010, 359–390). Based on SDT, students’ perceptions of voluntary autonomy, competence, and relatedness in academic majors fully mediate the relations between perceived faculty and peer support and major satisfaction (Schenkenfelder et al., 2020, 265–273). In addition, autonomous major choice motivation mediates the relation between autonomy-supportive parenting and academic major satisfaction, and controlled major choice motivation mediates the association between controlling parenting and academic major satisfaction (Nerona, 2021, 205–220).

Previous research provides a good foundation for this paper, but most of the analysis focuses on the impact of in-school experiences or training on satisfaction, and the basic and decisive role of the voluntary choice of college entrance examination is neglected. Previous literature has not sufficiently considered the impact of various admission characteristics on student satisfaction with college, such as whether the student is admitted to their first choice, whether it is their own choice, and whether there is an opportunity to choose their major again. Secondly, previous research has not considered the endogeneity problem caused by self-selection bias or omitted variables, which may result in overestimation or underestimation of the impact of admission characteristics on satisfaction. In addition, current research conclusions are difficult to provide specific policy recommendations and guidance for college entrance examination reform or college reform because they only generally analyze the factors affecting student satisfaction and do not specifically analyze which dimensions of student satisfaction are affected by which factors.

### 2.3. Research questions and hypotheses

Based on the above theory and literature analysis, this paper proposes the research questions and corresponding research hypotheses:

Question 1: How is the relationship between students’ admitted characteristics and students’ college satisfaction?

The admission characteristics referred to in this study include "whether admitted to first choice major," "whether admitted to first choice school," "freedom in choosing high school aspirations," and "admission through liberal arts recruitment." Based on the above theoretical foundation and literature review, the paper argues that students who are admitted to their first choice have higher levels of satisfaction and thus higher levels of satisfaction. Similarly, students who choose their own high school aspirations have more autonomy, stick to their own interests in learning, and may also have higher levels of satisfaction. Additionally, students who are admitted through the liberal arts recruitment process may have higher levels of satisfaction due to having more time and opportunities to choose their subsequent major. As a result, this paper proposes corresponding research hypotheses.

*H1.a*: Students’ college satisfaction is positively and significantly affected by the first-choice college.

*H1.b*: Students’ college satisfaction is positively and significantly affected by the first-choice major.

*H1.c*: Students’ college satisfaction is positively and significantly affected by the voluntary autonomy.

*H1.d*: Students’ college satisfaction is positively and significantly affected by Classified recruitment.

Question 2: Is there any difference in the influence of the admitted characteristics of different groups of students on students’ college satisfaction?

The impact of admission characteristics on the satisfaction of students in different types of institutions may vary. Students in "211 project" colleges may have higher satisfaction than those not admitted to their first choice major, as they are provided with better living conditions, academic atmosphere, and professional resources. However, the effect of being admitted to the first choice major on satisfaction may not be as obvious among students in non "211 project" schools. Similarly, the autonomy in choosing admission preferences and the admission method through broad category recruitment also result in higher satisfaction among students in "211 project" colleges. Therefore, the corresponding research hypothesis of this paper is proposed.

*H2.a*: Students’ college satisfaction in “Project 211” colleges is affected more positively and significantly by the first-choice college.

*H2.b*: Students’ college satisfaction in “Project 211” colleges is affected more positively and significantly by the first-choice major.

*H2.c*: Students’ college satisfaction in “Project 211” colleges is affected more positively and significantly by voluntary autonomy.

*H2.d*: Students’ college satisfaction in “Project 211” colleges is affected more positively and significantly by Classified recruitment.

Secondly, there may be differences in the impact of admitted characteristics on students’ college satisfaction in sciences and humanities. Since science students are more professional, the level of their major rather than the ranking of their school has a greater impact on their college satisfaction. Therefore, compared with students of humanities, "being admitted by their first-choice major" may have a greater impact on the college satisfaction of science students, while "being admitted by their first-choice college" has a relatively smaller impact on the college satisfaction of science students. In addition, science students have more major categories and more choices, while students of humanities have relatively few categories, so "voluntary autonomy" may have a greater impact on the college satisfaction of science students. However, "Classified recruitment" also enables both students of sciences and humanities. to have the opportunity to choose majors again, so the influence of " Classified recruitment " on their college satisfaction may not be different.

*H2.e*: Sciences students’ college satisfaction is affected less positively and significantly by the first-choice college.

*H2.f*: Sciences students’ college satisfaction is affected more positively and significantly by the first-choice major.

*H2.g*: Sciences students’ college satisfaction is affected more positively and significantly by the voluntary autonomy.

*H2.h*: There is no significant difference in the impact of Classified recruitment on the college satisfaction of students in sciences and humanities.

### 2.4. The innovation of this study

The innovation of this study is as follows. First, in terms of the measure of the independent variable, as former studies only take professional preferences into consideration when analyzing the admitted characteristics of college entrance examinations, the paper constructs a more comprehensive index to measure the admitted characteristics of college entrance examinations, including the first choices of colleges, independent selection of majors, and college admission routes. Second, in terms of measures of dependent variables, while overall satisfaction is generally used as the dependent variable in former studies, the paper expands the measurement of students’ college satisfaction, which is divided into academic satisfaction and nonacademic satisfaction. The former refers to satisfaction related to teaching, scientific research and courses, and the latter refers to interpersonal relationships. Third, in terms of research methods, there are endogeneity problems in former studies, and the accuracy of empirical estimation needs further improvement. Accordingly, the propensity score matching (PSM) method is adopted partly to solve endogeneity problems and obtain more accurate estimates. Fourth, in terms of heterogeneity analysis, whereas previous studies mainly focus on differences among students in different grades, the paper further explores the heterogeneity in student groups of different types of colleges.

## 3. Methods

### 3.1. Data sources and variable selection

All data result from the “Beijing College Student Panel Survey” (BCSPS) project. Respondents are full-time undergraduates enrolled in the fall of 2006 and 2008 and from public colleges under the direct leadership of the Ministry of Education, other central ministries, and the Beijing government. On this basis, provided by the Beijing Municipal Education Commission, the database of students enrolled in the fall of 2006 and 2008 is taken as the sampling frame. Various sampling methods—stratified sampling, multistage sampling, and probability-to-scale (PPS) sampling—are adopted. Eventually, 10,684 students from 15 colleges in Beijing are drawn as samples. This paper deletes samples with singular values and missing key variables such as register changes (withdrawal, extended term suspension, school resumption), admitted characteristics, and parents’ education attainment. Finally, 10,111 samples are extracted (Liu et al., 2019; Gao et al., 2022, 292–300; Liu X. et al., 2022, 1,481–1,487; Luo et al., 2022; Zhang et al., 2022).

For variable selection, this paper starts from the three perspectives of theory, science, and feasibility, on the basis of SDT, draws on the experience of previous literature and combines the availability of survey data, and selects “college entrance examination admission characteristic” as the core independent variable. Including the result of admission (whether the current college and major is the first choice), the process of admission (voluntary autonomy), the way of admission (classified recruitment or non-classified recruitment); “overall satisfaction,” “academic satisfaction” and “non-academic satisfaction” were selected as dependent variables. “Individual features,” “family background,” “experience in colleges,” “types of colleges” and other factors that also affect students’ college satisfaction are selected as control variables to solve the problem of missing variables.

### 3.2. Variable measurement and data description

Tables 1, 2, respectively, show the measurement method of specific variables and basic descriptive statistics, respectively. Therein, as for basic descriptive statistics, in terms of admitted characteristics, 16.74% of respondents are studying in colleges that are not their first choices; for 36.90% of respondents, their current majors are not their first choices; 52.18% of the respondents say that they are greatly influenced by parents, teachers and friends when applying for colleges and majors. In terms of individual features, males account for 51.84%; urban respondents account for 55.98%; students in sciences account for 99.24%; and students from “Project 211” colleges and key high schools account for 69.99 and 88.57%, respectively.

**Table 1:** Measurement of specific variables.

Types of variables	Definitions of variables		Measurement of variables
Independent variable	Admitted characteristics		Whether the current college is the first choice?(Yes = 1, No = 0)
			Whether the current major is the first choice? (Yes = 1, no = 0)
			Voluntary autonomy (by oneself = 1, affected by others = 0)
			Admission routes (classified recruitment = 1, non-classified recruitment = 0)
Dependent variables	Overall satisfaction		Overall satisfaction (1-10points, 1 = highly dissatisfied, 10 = highly satisfied)
	Academic satisfaction		Overall satisfaction on academic factors (1–10points, 1 = highly dissatisfied, 10 = highly satisfied)
	Nonacademic satisfaction		Overall satisfaction on nonacademic factors (1–10points, 1 = highly dissatisfied, 10 = highly satisfied)
Controlled variables	Individual features	Gender	(Male = 1, female = 0)
		Registered residence	(Urban =1, rural = 0)
		Grade	(Freshman = 1, sophomore = 2, junior = 3, senior = 4)
		Types of high school	(Key high school = 1, regular high school = 0)
		Division of sciences and humanities in high school	(Humanities = 1, sciences = 0)
		Reattendance of college entrance examination	(Yes = 1, no = 0)
	Family background	Parents’ educational attainment	No formal education = 1, primary school = 2, junior high school = 3, high school = 4, vocational/technical school = 5, technical secondary school = 6, junior college = 7, undergraduate = 8, postgraduate and above = 9
		Household income	Logarithm of the annual income
	Experiences in colleges	academic Achievements(scores)	Ranks in class (rank/class size)
		Teacher-student relationship and the relationship with classmates	Intimacy with classmates (1–10points, 1 = highly alienated, 10 = highly intimated)
			Intimacy with roommates (1–10points, 1 = highly alienated, 10 = highly intimated)
			Intimacy with teachers (1–10points, 1 = highly alienated, 10 = highly intimated)
		Academic efficacy	(Academic Efficacy Subscale in PALS，1–5points)
		learning motivation	Achievement Goal Framework by Elliot and McGregor (2001) (external learning motivation, 1–5points)
			Achievement Goal Framework by Elliot and McGregor (2001) (internal learning motivation, 1–5points)
		Club participation	Hours devoted to club participation per semester
	Types of colleges	“Project 211”colleges = 1, others = 0	

**Table 2:** Basic descriptive statistics (%).

		Total	Choice of college		Choice of major		Voluntary autonomy		Admission routes	
			First-choice	Nonfirst-choice	First-choice	Nonfirst-choice	By oneself	By others	Classified recruitment	Non-classified recruitment
Gender	Female	48.16	39.35	8.81	30.06	18.10	21.25	26.91	0.50	47.65
	Male	51.84	43.92	7.93	33.04	18.80	26.58	25.27	0.25	51.59
Registered residence	Rural	44.02	36.93	7.09	27.72	16.30	21.61	22.41	0.18	43.84
	Urban	55.98	46.34	9.64	35.38	20.60	26.22	29.76	0.58	55.40
Types of colleges	Non “Project 211” colleges	30.01	18.35	11.66	18.50	11.51	13.68	16.33	0.15	29.86
	“Project 211” colleges	69.99	64.92	5.07	44.60	25.39	34.15	35.84	0.61	69.38
Types of high school	Regular high school	11.43	8.96	2.47	7.95	3.48	5.25	6.18	0.13	11.31
	Key high school	88.57	74.31	14.26	55.15	33.42	42.58	45.99	0.63	87.94
Division of sciences and humanities in high school	Humanities	0.76	0.71	0.0	0.45	0.30	0.61	0.15	0.10	0.66
	Sciences	99.24	82.56	16.68	62.65	36.60	51.56	47.68	0.66	98.59

Table 3 presents the average satisfaction of different groups on whether the current major is the first choice. Respondents whose current majors are their first choices have higher satisfaction than those who are not. The satisfaction and academic satisfaction of males are higher than those of females, while the nonacademic satisfaction of females is higher than that of males. The satisfaction of rural students whose majors are their first choices is slightly higher than that of urban students, but it is almost the same when current majors are not their first choices. Students from “Project 211” colleges are more satisfied than those who are not, even when current majors are not their first choices. The satisfaction of students from key middle schools is higher than that of those not.

**Table 3:** Average satisfaction of different groups with the first-choice major.

		The first-choice major			Nonfirst-choice major		
		Overall satisfaction	Academic satisfaction	Nonacademic satisfaction	Overall satisfaction	Academic satisfaction	Nonacademic satisfaction
Gender	Female	6.642	6.666	6.565	6.500	6.512	6.462
	Male	6.700	6.767	6.469	6.510	6.530	6.398
Registered residence	Rural	6.717	6.770	6.540	6.522	6.539	6.467
	Urban	6.638	6.680	6.497	6.510	6.518	6.437
Types of colleges	Non “Project 211” colleges	6.112	6.124	6.074	5.982	5.966	6.044
	“Project 211” colleges	6.906	6.966	6.698	6.734	6.774	6.604
Types of high school	Regular high school	6.256	6.270	6.212	6.135	6.146	6.097
	Key high school	6.866	6.928	6.657	6.646	6.672	6.562
Division of sciences and humanities in high school	Humanities	6.734	6.745	6.699	6.647	6.648	6.662
	Sciences	6.646	6.708	6.438	6.454	6.483	6.358

### 3.3. Advantages and basic steps of propensity score matching

The advantage of PSM is that it can alleviate the problems of self-selection and missing variables and obtain a more accurate estimation. The net causal effect is inaccessible to conventional multiple linear regression. The core independent variable of this paper is college entrance examination admission characteristics. Taking the independent variable “whether the current college is the first choice” as an example, it is not exogenous to a large extent but can be independently chosen, and it may be affected by variables such as family background, parents’ educational attainment, types of high school, and reattendance of college entrance examinations, which also affect students’ college satisfaction. If differences in satisfaction of student groups between first-choice and nonfirst-choice admission are directly compared, there may be a greater bias due to the endogenous problem caused by self-selection bias and missing variables. Therefore, first, in order to solve the problem of missing variables, we need to control the influence of these factors. The establishment of multiple regression model is one of the commonly used methods, but before setting the multiple regression model, researchers should clarify the functional relationship between X and Y. Otherwise, functional form misspecification (FFM) will occur, resulting in biased estimation coefficients. The advantage of PSM is that it does not rely on explicit model-setting assumptions, thus avoiding estimation bias due to model-setting bias. Second, in order to solve the self-selection problem, PSM will be used in this paper to match each student who is “admitted by the first choice” (intervention group) with a similar student who is “not admitted by the first choice” (control group). At this time, it can be considered that the allocation of “first choice” (intervention variable) among students is random. The difference in satisfaction between the two types of students is mainly caused by the intervention variable “whether they are the first choice or not,” thus alleviating the self-selection problem and obtaining a more accurate estimation.

Propensity score matching generally consists of the following four steps. First, propensity score estimation. We identify a number of covariants that can affect both first-choice admission and students’ college satisfaction. With “whether the current college and major are first choices” as the dependent variable, a logit/probit model is set to calculate each student’s propensity score, namely, the probability of being admitted by their first choices. Second, propensity score matching. We adopt the 1-to-1 nearest neighbor matching method to match the student’s propensity score, namely, to match each student who is admitted by his first choice with one who is not. Both have similar propensity scores. Third, balance test. One way is to look at the distribution of propensity scores before and after matching. The closer the distribution of propensity scores between the intervention group and the control group after matching, the smaller the gap between the two types of students. The second method is to estimate the difference between the two groups of students in each covariable. If the difference is not significant, it means that there is no obvious difference between the two groups of students. Finally, causal effect estimation. Because the propensity score matching method is used to eliminate the self-selection problem of “whether to be admitted as the first choice,” it can be concluded that the distribution of the intervention variable, that is, “whether the current college and major are first choices,” among students is random, and differences in satisfaction mainly result from the intervention variable of “whether it is the first choice.”

### 3.4. Empirical model setting

Therefore, the following empirical model is set, and the weight is adopted for regression analysis. *i* and *f* in the model represent individual and fixed effect, respectively:


$$\begin{array}{l} Satisfaction_{i}=\alpha +\beta_{1}*Major\_first_{i}+\beta_{2}*School\_first_{i}\\ +\beta_{3}*Autonomy_{i}+\beta_{4}*Enroll_{i}+\beta_{5}*Family_{i}\\ +\beta_{6}*Performance_{i}+Grade_{f}+Year_{f}\\ +\;College_{f}+Track_{f}+Subject_{f}+\mu_{i} \end{array}$$

$Satisfaction_{i}$ represents the overall satisfaction/academic satisfaction/nonacademic satisfaction; the independent variable of $Major\_first_{i}$ indicates whether the current major is the first choice, which is the major concern of this paper; $School\_first_{i}$ indicates whether the current college is the first choice; $Autonomy_{i}$ indicates the student’s voluntary autonomy; $Enroll_{i}$ indicates whether the student’s admission route is classified recruitment; $Family_{i}$ represents variables of family background such as income and parents’ educational attainment; $Performance_{i}$ indicates the student’s performance in colleges, such as academic achievements, self-efficacy, learning motivation, teacher-student relationship, etc.; $Grade_{f}$ 、 $Year_{f}、College_{f}、Track_{f}、Subject_{f}$ represent the fixed effects of grade, year of enrollment, types of colleges, division of sciences and humanities in high school, and discipline categories in turn. Adding fixed effect mainly plays a role in controlling related missing variables. For example, students in the same grade, the same year of entry, the same college type, the same college entrance examination subject or the same subject category may have similar characteristics. In order to exclude the influence of these characteristics on students’ college satisfaction that has not been observed, we use the fixed effect model for estimation.

## 4. Findings

### 4.1. Propensity score matching process

The results in Table 4 show that there are significant differences in characteristics before enrollment between first-choice students and nonfirst-choice students, and these variables also affect students’ college satisfaction. To account for this, the propensity score matching method is used to alleviate the endogeneity problem. Logit regression results in Table 5 show the significant impact of variables other than “annual household income” and “reattendance of college entrance examination” on the variable of “whether the current college is the first choice.” The 1-to-1 nearest neighbor matching method is adopted to match the propensity scores of the experimental group (first-choice admission) with the control group (nonfirst-choice admission) 2. Then, the standard deviation for most covariants between the experimental group and the control group drops to within 10%, and there are no longer significant differences.

**Table 4:** Differences in characteristics between first-choice students and nonfirst-choice students.

	Nonfirst-choice major	First-choice major	*D*-value	Nonfirst-choice college	First-choice college	*D*-value
	(M/S.D.)	(M/S.D.)	(Coefficient/S.E)	(M/S.D.)	(M/S.D.)	(Coefficient/S.E)
Overall satisfaction	6.466	6.694	−0.227^***^	6.082	6.716	−0.634^***^
	(1.739)	(1.808)	(0.037)	(1.616)	(1.800)	(0.047)
Academic satisfaction	6.497	6.740	−0.243^***^	6.110	6.759	−0.650^***^
	(1.837)	(1.953)	(0.039)	(1.699)	(1.936)	(0.051)
Nonacademic satisfaction	6.343	6.466	−0.123^**^	5.974	6.511	−0.537^***^
	(1.970)	(2.004)	(0.041)	(1.879)	(2.002)	(0.053)
Nation	0.879	0.890	−0.011	0.907	0.881	0.026^**^
	(0.327)	(0.314)	(0.007)	(0.291)	(0.324)	(0.008)
Registered residence	0.567	0.571	−0.003	0.590	0.565	0.024
	(0.495)	(0.495)	(0.010)	(0.492)	(0.496)	(0.013)
Father’s educational attainment	5.491	5.541	−0.050	5.305	5.566	−0.261^***^
	(2.236)	(2.287)	(0.047)	(2.222)	(2.275)	(0.060)
Mother’s educational attainment	5.075	5.025	0.050	4.988	5.054	−0.067
	(2.258)	(2.259)	(0.047)	(2.207)	(2.269)	(0.060)
Types of high school	0.714	0.682	0.032^***^	0.668	0.699	−0.031^*^
	(0.452)	(0.466)	(0.009)	(0.471)	(0.459)	(0.012)
Scores of college entrance examination	576.300	577.735	−1.435	538.388	585.067	−46.679^***^
	(74.220)	(83.713)	(1.660)	(69.842)	(80.009)	(2.093)
Reattendance of college entrance examination	0.165	0.185	−0.020^*^	0.149	0.183	−0.034^***^
	(0.371)	(0.388)	(0.008)	(0.356)	(0.387)	(0.010)
	8.928	8.902	0.025	9.014	8.891	0.123
	(3.662)	(3.671)	(0.076)	(3.614)	(3.678)	(0.098)
Logarithm of annual household income	3,743	6,368	10,111	1,696	8,415	10,111

**Table 5:** Estimation and matching results of propensity score (taking “whether the current college is the first choice” as an example).

Variable	Logit regression	Before matching	Mean		SD (%)	Deviation reduction (%)	*T*-test	
		After matching	Control group	Experimental group			*t*	*p* > *t*
Nation	−0.147^***^	U	0.90632	0.88108	8.2	90.5	2.96	0.003
	(0.055)	M	0.90632	0.90871	−0.8		−0.24	0.812
Registered residence	−0.024	U	0.58711	0.5639	4.7	87.1	1.75	0.08
	(0.040)	M	0.58711	0.58413	0.6		0.18	0.861
Father’s educational attainment	0.031^***^	U	5.2977	5.5571	−11.5	94	−4.28	0
	(0.011)	M	5.2977	5.2822	0.7		0.2	0.84
mother’s educational attainment	−0.006	U	4.9827	5.0456	−2.8	64	−1.04	0.298
	(0.012)	M	4.9827	4.96	1		0.3	0.766
Types of high school	−0.122^***^	U	0.66885	0.70059	−6.8	69.9	−2.58	0.01
	(0.039)	M	0.66885	0.6784	−2.1		−0.59	0.556
Reattendance of college entrance examination	0.145^***^	U	0.14797	0.18361	−9.6	98.3	−3.48	0
	(0.045)	M	0.14797	0.14857	−0.2		−0.05	0.961
Annual household income	0.000	U	62,091	66,642	−1.6	31.9	−0.48	0.629
	(0.000)	M	62,091	58,991	1.1		0.86	0.391

**Figure 1:**
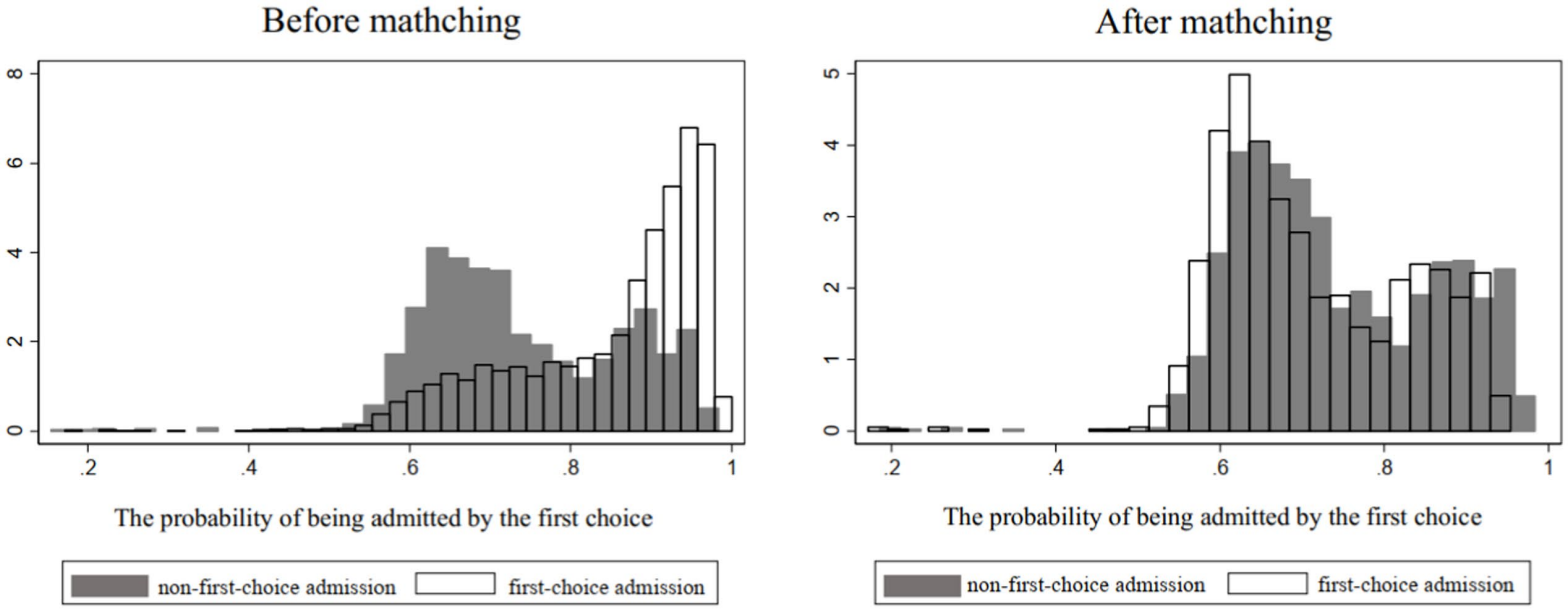
Samples before and after matching.

Meanwhile, Figure 1 shows that there are differences between the first-choice group and nonfirst-choice group before matching, but there is enough overlap (samples of common value) for matching. The comparability of the two groups was significantly improved after matching.

### 4.2. Overall impact of the admitted characteristics of college entrance examinations on students’ college satisfaction

Table 6 presents the impact of variables of the total sample on overall college satisfaction, academic satisfaction, and nonacademic satisfaction. Meanwhile, the regression results of OLS (Ordinary Least Squares) and PSM are also compared, indicating that OLS overestimates the impact of the first choice on satisfaction promotion, whereas PSM effectively alleviates the estimation bias caused by selection bias to some extent.

**Table 6:** Regression results of the total sample.

		Overall satisfaction		Academic satisfaction		Nonacademic satisfaction	
		OLS	PSM	OLS	PSM	OLS	PSM
Admitted characteristics	The first-choice major	0.164^***^	0.108^*^	0.181^***^	0.138^**^	0.066	0.001
		(0.037)	(0.060)	(0.039)	(0.063)	(0.042)	(0.070)
	The first-choice college	0.282^***^	0.266^***^	0.275^***^	0.237^***^	0.279^***^	0.360^***^
		(0.049)	(0.063)	(0.052)	(0.067)	(0.056)	(0.073)
	Choosing major independently	0.071^**^	0.114^**^	0.077^**^	0.121^**^	0.046	0.100
		(0.034)	(0.057)	(0.037)	(0.060)	(0.039)	(0.066)
	Classified recruitment	0.249	0.117	0.279	0.304	0.202	−0.529
		(0.245)	(0.434)	(0.246)	(0.396)	(0.321)	(0.668)
Individual features	Male	−0.151^***^	−0.187^***^	−0.165^***^	−0.218^***^	−0.120^***^	−0.101
		(0.040)	(0.065)	(0.043)	(0.069)	(0.045)	(0.076)
	Han	0.118^**^	0.093	0.124^**^	0.087	0.034	0.034
		(0.058)	(0.103)	(0.062)	(0.108)	(0.064)	(0.117)
	Urban area	−0.087^**^	−0.069	−0.083^*^	−0.015	−0.123^**^	−0.200^**^
		(0.043)	(0.069)	(0.046)	(0.074)	(0.048)	(0.081)
	Key high school	0.166^***^	0.191^**^	0.178^***^	0.198^**^	0.148^**^	0.236^**^
		(0.059)	(0.084)	(0.063)	(0.088)	(0.065)	(0.097)
	Reattendance of college entrance examination	−0.064	−0.071	−0.068	−0.046	−0.065	−0.102
		(0.047)	(0.081)	(0.050)	(0.085)	(0.052)	(0.094)
	Score of college entrance examination	0.003^***^	0.001^**^	0.003^***^	0.002^***^	0.002^***^	0.001
		(0.000)	(0.001)	(0.000)	(0.001)	(0.000)	(0.001)
	Father’s educational attainment	0.023^**^	0.016	0.026^**^	0.020	0.001	0.014
		(0.012)	(0.020)	(0.012)	(0.021)	(0.013)	(0.022)
	Mother’s educational attainment	−0.025^**^	−0.063^***^	−0.024^*^	−0.065^***^	−0.021	−0.055^**^
		(0.012)	(0.021)	(0.013)	(0.022)	(0.014)	(0.023)
	Annual household income	−0.028	−0.053^*^	−0.027	−0.049^*^	−0.031^*^	−0.062^**^
		(0.017)	(0.027)	(0.018)	(0.028)	(0.019)	(0.028)
Experience in college	Intrinsic learning motivation	0.041^***^	0.043^***^	0.041^***^	0.044^***^	0.035^***^	0.028^***^
		(0.005)	(0.008)	(0.005)	(0.008)	(0.005)	(0.009)
	Extrinsic learning motivation	−0.004	−0.000	−0.005	−0.002	0.002	0.015
		(0.005)	(0.009)	(0.006)	(0.009)	(0.006)	(0.010)
	Ranks in academic achievements	−0.022^***^	−0.046^***^	−0.027^***^	−0.056^***^	−0.011	−0.018
		(0.008)	(0.014)	(0.009)	(0.014)	(0.009)	(0.016)
	Self-efficacy	0.142^***^	0.195^***^	0.145^***^	0.218^***^	0.117^***^	0.122^*^
		(0.038)	(0.063)	(0.041)	(0.067)	(0.043)	(0.071)
	Intimacy with classmates	0.538^***^	0.499^***^	0.548^***^	0.529^***^	0.511^***^	0.409^**^
		(0.086)	(0.135)	(0.091)	(0.141)	(0.099)	(0.162)
	Intimacy with roommates	0.413^***^	0.519^***^	0.370^***^	0.469^***^	0.574^***^	0.687^***^
		(0.053)	(0.088)	(0.057)	(0.094)	(0.060)	(0.102)
	Intimacy with teachers	0.390^***^	0.518^***^	0.304^***^	0.442^***^	0.705^***^	0.804^***^
		(0.066)	(0.110)	(0.071)	(0.116)	(0.073)	(0.120)
	Hours devoted to club participation	0.007^***^	0.008^***^	0.006^***^	0.006^**^	0.009^***^	0.010^***^
		(0.002)	(0.003)	(0.002)	(0.003)	(0.002)	(0.004)
Observation		9,788	3,277	9,806	3,281	9,785	3,276
*R* ^2^		0.163	0.183	0.154	0.178	0.127	0.148

(1). ^***^Denotes the significance of 0.01; ^**^denotes the significance of 0.05; ^*^denotes the significance of 0.10. Values in brackets are standard errors. Similarly, hereinafter. (2). All models are set with grades, year of enrollment, types of colleges, discipline categories, and division of sciences and humanities in high school under control.

The empirical research shows that all three types of satisfaction are affected positively and significantly by the first-choice college (Hypothesis 1.a is tested), while first-choice major has no significant impact on nonacademic satisfaction, indicating that students still attach great importance to the first-choice college. In contrast, first-choice major has only a minor impact on overall satisfaction and has nonsignificant impact on college nonacademic satisfaction (Hypothesis 1.b is partially verified). In addition, the variable “choosing major independently” also contributes to the promotion of overall satisfaction and academic satisfaction, and its influence on overall satisfaction is even greater than that of the first-choice major (Hypothesis 1.c is tested). Choosing a major according to classified categories (that is, the major is not yet decided at the time of admission and will be determined after enrollment) has no significant effect on satisfaction promotion (Hypothesis 1.d is not tested).

Meanwhile, empirical research also shows that stronger intrinsic learning motivation, higher academic achievements and stronger self-efficacy can greatly promote students’ college satisfaction, while extrinsic learning motivation has no notable influence. Moreover, the more intimate the relationship between students and their classmates and roommates, the higher overall satisfaction will be. This effect will even exceed that of the relationship with teachers, which is consistent with research findings by Yuheng et al. (2016). They also found that interpersonal relationships with peers and teachers are key factors in students’ college satisfaction.

### 4.3. Heterogeneous influence of the characteristics of college entrance examinations on students’ college satisfaction

Table 7 shows the influence of the characteristics of college entrance examinations on the satisfaction of different student groups. In terms of the heterogeneity of college types, the satisfaction of students in non “Project 211” colleges is significantly and positively affected by the first-choice college, while the first-choice major has no significant effect on students’ college satisfaction. However, for students in “Project 211″ colleges, their satisfaction is positively affected by both the first-choice college and the first-choice major, and only nonacademic satisfaction is not affected by the first-choice major (Hypothesis 2.a & 2.b is partially verified). In addition, “voluntary autonomy” can promote the overall satisfaction and academic satisfaction of students in non “Project 211″ colleges and has a positive impact on the nonacademic satisfaction of students in “Project 211″ colleges (Hypothesis 2.c is not tested). Classified recruitment has a positive effect on all three types of satisfaction in “Project 211″ colleges (Hypothesis 2.d is tested) but has a negative impact on the nonacademic satisfaction of students in non “Project 211″ colleges.

**Table 7:** The influence of college entrance examination admission characteristics on students’college satisfaction in different groups.

Types of colleges		Non “Project 211” colleges (*N* = 2039)			“Project 211” colleges (*N* = 1,241)		
		Overall satisfaction	Academic satisfaction	Nonacademic satisfaction	Overall satisfaction	Academic satisfaction	Nonacademic satisfaction
Admitted characteristics	The first-choice major	0.007	0.034	−0.073	0.221^**^	0.263^**^	0.062
		(0.076)	(0.079)	(0.090)	(0.101)	(0.109)	(0.117)
	The first-choice college	0.228^***^	0.188^**^	0.341^***^	0.257^**^	0.250^**^	0.319^***^
		(0.081)	(0.085)	(0.093)	(0.104)	(0.114)	(0.122)
	Voluntary autonomy	0.125^*^	0.142^*^	0.032	0.151	0.140	0.254^**^
		(0.072)	(0.075)	(0.086)	(0.099)	(0.106)	(0.113)
	Classified recruitment	−0.687	−0.320	−1.998^***^	2.117^***^	1.944^***^	2.755^***^
		(0.449)	(0.422)	(0.677)	(0.361)	(0.331)	(0.754)
Sciences/humanities		Humanities (*N* = 838)			Sciences (*N* = 2,443)		
		Overall satisfaction	Academic satisfaction	Nonacademic satisfaction	Overall satisfaction	Academic satisfaction	Nonacademic satisfaction
Admitted characteristics	The first-choice major	0.084	0.091	0.060	0.125^*^	0.157^**^	−0.009
		(0.136)	(0.144)	(0.150)	(0.068)	(0.072)	(0.081)
	The first-choice college	0.589^***^	0.604^***^	0.544^***^	0.165^**^	0.126	0.295^***^
		(0.135)	(0.142)	(0.150)	(0.073)	(0.079)	(0.086)
	Voluntary autonomy	0.030	0.053	−0.055	0.133^**^	0.135^*^	0.150^*^
		(0.132)	(0.139)	(0.147)	(0.065)	(0.069)	(0.077)
	Classified recruitment	−0.584	−0.617	−0.469	−0.023	0.326	−1.181
		(0.599)	(0.608)	(0.646)	(0.540)	(0.499)	(0.783)

In terms of the heterogeneity of students in sciences and humanities, it can be found that both the first-choice major and voluntary autonomy have a great and positive impact on science students (Hypothesis 2.f & 2.g is tested), while the first-choice college greatly impacts students of humanities (Hypothesis 2.e is tested). Possible reasons are that sciences students focus more on voluntary autonomy, while students of humanities attach more importance to types of colleges. Additionally, classified recruitment has no significant influence on the students’ college satisfaction in sciences and humanities (Hypothesis 2.h is tested).

### 4.4. The influence of cross-category adjustment on students’ college satisfaction

This paper further analyzes the student group whose current majors are not their first choices and divides the students’ current majors into corresponding fields of study in two ways. In one way, they are divided into the group of humanities and social sciences and the group of engineering, agriculture, and medical science. In other words, they are divided into seven subcategories: social sciences, liberal arts, sciences, agricultural science, agriculture, medical science, and management. If the first-choice major and the current major of nonfirst-choice students are divided into different categories according to the first sorting technique, they are classified into the cross-category class (cross-category = 1). If they are divided into the same category, then they are classified into the noncross-category class (noncross-category = 0). Similarly, there are cross-category classes and noncross-category classes in the second method. However, the difference is that the first-choice majors and their current majors of students who are classified into the cross-category class in the first method vary greatly, while the first-choice majors and current majors of students who are classified into the cross-category class in the second method actually have minor differences. Hence, the analysis mainly explores the current situation of satisfaction of students whose current majors are not their first choices in the case of different categories of current major and the first-choice major.

The empirical results in Table 8 indicate that both the larger cross-category class and the minor cross-category class have a significant negative impact on the academic satisfaction of the total sample, but the impact on nonacademic satisfaction is not significant. Moreover, the larger cross-category class has a significant negative impact on overall satisfaction, while the minor cross-category class does not. The regression results of the subsamples show that both the larger cross-category class and the minor cross-category class have a significant negative impact on students’ college satisfaction in “Project 211″ colleges but have no significant impact on students in non “Project 211″ colleges. In addition, the minor cross-category class has a significant negative impact on liberal arts students, while the larger cross-category class has a significant negative impact on science students.

**Table 8:** Impacts of the characteristics of college entrance examinations on the satisfaction of students whose current majors are not their first choices.

Total sample (*N* = 3,607)		Overall satisfaction		Academic satisfaction		Nonacademic satisfaction	
Admitted characteristics	The first-choice college	0.257^***^	0.255^***^	0.228^***^	0.226^***^	0.313^***^	0.314^***^
		(0.074)	(0.074)	(0.078)	(0.078)	(0.087)	(0.087)
	Voluntary autonomy	0.065	0.064	0.037	0.036	0.103	0.104
		(0.057)	(0.057)	(0.060)	(0.060)	(0.065)	(0.065)
	Classified recruitment	1.072^**^	1.022^**^	1.288^***^	1.216^**^	0.404	0.452
		(0.509)	(0.509)	(0.474)	(0.474)	(0.717)	(0.716)
	Larger cross-category class	−0.123^*^		−0.174^**^		0.097	
		(0.070)		(0.074)		(0.081)	
	Minor cross-category class		−0.098		−0.135^*^		0.052
			(0.067)		(0.071)		(0.077)
Non“Project 211”colleges (*N* = 1,108)		Overall satisfaction		Academic satisfaction		Nonacademic satisfaction	
Admitted characteristics	The first-choice major	0.199^*^	0.199^*^	0.148	0.149	0.383^***^	0.381^***^
		(0.118)	(0.118)	(0.122)	(0.122)	(0.136)	(0.136)
	Voluntary autonomy	0.164	0.164	0.145	0.147	0.221^*^	0.218^*^
		(0.108)	(0.108)	(0.113)	(0.113)	(0.125)	(0.125)
	Classified recruitment	−0.312	−0.329	0.020	−0.024	−1.479	−1.404
		(0.727)	(0.724)	(0.681)	(0.678)	(1.030)	(1.029)
	Larger cross-category class	−0.028		−0.074		0.132	
		(0.123)		(0.128)		(0.143)	
	Minor cross-category class		0.015		0.007		0.038
			(0.120)		(0.125)		(0.136)
“Project 211”colleges (*N* = 2,499)		Overall satisfaction		Academic satisfaction		Nonacademic satisfaction	
Admitted characteristics	The first-choice college	0.171^*^	0.165^*^	0.146	0.139	0.151	0.153
		(0.100)	(0.100)	(0.106)	(0.106)	(0.120)	(0.120)
	Voluntary autonomy	0.051	0.048	0.014	0.009	0.105	0.106
		(0.068)	(0.068)	(0.073)	(0.073)	(0.078)	(0.078)
	Classified recruitment	2.570^***^	2.514^***^	2.638^***^	2.571^***^	2.533^***^	2.557^***^
		(0.244)	(0.238)	(0.259)	(0.255)	(0.280)	(0.280)
	Larger cross-category class	−0.172^*^		−0.214^**^		0.065	
		(0.089)		(0.096)		(0.104)	
	Minor cross-category class		−0.127		−0.161^*^		0.042
			(0.086)		(0.091)		(0.100)
Liberal arts students (*N* = 842)		Overall satisfaction		Academic satisfaction		Nonacademic satisfaction	
Admitted characteristics	The first-choice major	0.332^*^	0.330^*^	0.339^*^	0.337^*^	0.272	0.275
		(0.184)	(0.184)	(0.193)	(0.193)	(0.212)	(0.211)
	Voluntary autonomy	−0.075	−0.071	−0.146	−0.142	0.071	0.074
		(0.135)	(0.134)	(0.144)	(0.144)	(0.154)	(0.154)
	Classified recruitment	4.013^***^	3.985^***^	4.146^***^	4.116^***^	3.534^***^	3.491^***^
		(0.921)	(0.913)	(0.964)	(0.956)	(0.878)	(0.876)
	Larger cross-category class	0.157		0.177		0.357	
		(0.310)		(0.350)		(0.297)	
	Minor cross-category class		−0.315^**^		−0.297^*^		−0.267
			(0.151)		(0.163)		(0.170)
Science students (*N* = 2,765)		Overall satisfaction		Academic satisfaction		Nonacademic satisfaction	
Admitted characteristics	The first-choice college	0.190^**^	0.189^**^	0.147^*^	0.144	0.277^***^	0.279^***^
		(0.084)	(0.084)	(0.089)	(0.089)	(0.099)	(0.099)
	Voluntary autonomy	0.065	0.064	0.041	0.040	0.099	0.101
		(0.064)	(0.064)	(0.068)	(0.068)	(0.073)	(0.073)
	Classified recruitment	1.120^**^	1.046^*^	1.363^***^	1.272^**^	0.347	0.351
		(0.558)	(0.557)	(0.521)	(0.522)	(0.775)	(0.770)
	Larger cross-category class	−0.149^**^		−0.202^***^		0.067	
		(0.074)		(0.078)		(0.086)	
	Minor cross-category class		−0.057		−0.107		0.111
			(0.078)		(0.083)		(0.089)

## 5. Conclusion and discussion

Based on the follow-up survey data of undergraduates in 15 colleges in Beijing, this paper adopts the propensity score matching method to analyze the influence of the admitted characteristics of college entrance examinations—whether the current college and major are first choices and whether to make a choice independently—on undergraduates’ satisfaction. It further explores differences between student groups at different institutional levels and of different disciplines (liberal arts or sciences) and the impact of the span between the first-choice major and current major on students’ college satisfaction of students not admitted by the first choice. According to the empirical findings, the following five conclusions can be drawn:

First, both the first-choice college and voluntary autonomy have a significant impact on students’ college satisfaction. Therefore, the first-choice college has a significant and positive impact on all three types of satisfaction, while the first-choice major has no significant impact on nonacademic satisfaction, indicating that students still attach great importance to whether they are admitted by ideal colleges rather than preferred majors. Voluntary autonomy can greatly improve overall satisfaction and academic satisfaction, and its impact on overall satisfaction is even greater than that of the first-choice major, which indicates that students focus more on voluntary autonomy. This indicates that during the voluntary selection process of the entrance exam, students have both the desire for autonomous choice and the aspiration to enter a prestigious university, which is consistent with previous research findings Ding, 2019. In comparison, the impact of being admitted to the first-choice major they selected on their satisfaction is minimal.

Second, peer relations at the undergraduate level will greatly promote students’ college satisfaction. The more intimate the relationship between students and their classmates and roommates, the higher the overall satisfaction will be. This effect even exceeds that of intimacy with teachers. This is similar to previous research findings that students are more likely to have deeper communication with their peers who share the same living environment and growth stage. On the other hand, fresh students who are away from their families and in an unfamiliar environment have not yet formed a stable network of relationships, and they mainly engage in learning and social activities with similar peers. When facing adaptive problems such as learning and life, they tend to seek advice from their peers and quickly solve problems. The role of peer interaction even surpasses the influence of teacher-student interaction, and universities should pay attention to and give full play to the important role of student peers in the student development process.

Third, there are differences in students’ college satisfaction at the institution level. Students’ college satisfaction in non “Project 211″ colleges is positively affected by the first-choice college but not by the first-choice major. In contrast, students’ college satisfaction in “Project 211″ colleges is positively affected by both the first-choice college and the first-choice major. The reason is that, on the one hand, students in non “Project 211″ colleges are more concerned about whether they have been admitted to higher-level colleges, and on the other hand, they have more choices due to their higher scores on the college entrance examination. In addition, classified recruitment promotes all three types of satisfaction in “Project 211″ colleges, but it negatively impacts nonacademic satisfaction in non“Project 211″ colleges, which indicates that classified recruitment is probably not suitable for all colleges and universities.

Fourth, different admitted characteristics have different impacts on the students of liberal arts and sciences. The first-choice major and voluntary autonomy greatly and positively impact science students, while the first-choice college has a greater impact on liberal arts students. It follows then that science students pay more attention to voluntary autonomy and majors, while liberal arts students attach great importance to colleges. The possible reason is that science students are more professional, so they pay more attention to whether the current major is the first choice, while liberal arts students pay more attention to the brands of colleges in employment. The possible reason is that in the labor market, students in liberal arts majors are more replaceable due to their weaker professional skills. However, university rankings can compensate for and mitigate this employment disadvantage to some extent, leading to liberal arts students being more focused on the level of the university rather than the type of major when filling out college entrance examination plans. This also implies that in future policy reforms, universities should both enhance the core competitiveness and professional literacy of liberal arts students from an academic perspective and reduce discrimination against liberal arts majors from an employment perspective.

Fifth, adjustments of both large-span class and small-span class have a significant and negative impact on academic satisfaction but have no significant impact on nonacademic satisfaction. The regression results of subsamples show that both large-span and small-span adjustments have a significant and negative impact on students’ college satisfaction in “Project 211″ colleges but have no significant impact on students in non “Project 211″ colleges. Small-span class adjustment has a significant and negative impact on liberal arts students, while large-span class adjustment has a significant and negative impact on science students.

Based on the findings above, this paper proposes the following three suggestions.

First, students should follow their inner voice when applying for colleges and majors and rationally perceive the “halo” effect of famous colleges. Voluntary autonomy can largely improve students’ college satisfaction. College candidates are prone to be influenced by high school teachers and parents when filling out application forms. Blindly following others’ advice rather than the inner voice, students will generate persistent dissatisfaction after enrollment and thereafter will affect their academic achievements and physical and psychological health in colleges. Meanwhile, whether the current college is the first choice is a key factor affecting students’ college satisfaction in non “Project 211″ colleges. Students should reasonably recognize the “halo” effect of famous colleges and continuously strengthen their professional disciplines and skills to compensate for the dissatisfaction caused by nonfirst-choice colleges.

Second, colleges and universities should emphasize the training and cultivation of students’ interpersonal communication with classmates and teachers. Students’ college satisfaction is a key indicator to measure the quality of talent training. Colleges and universities should focus on cultivating self-efficacy and intrinsic learning motivation in terms of curriculum setting, teaching methods, and scientific research activities to improve students’ college satisfaction. At the same time, colleges and universities should vigorously promote communication and exchanges between students and their classmates, roommates, and teachers and pay attention to peer relationships in club activities and accommodation management. A good peer relationship and teacher-student relationship are beneficial to students’ college satisfaction and facilitate academic prospects and career choices.

Finally, the administrative department of education and colleges should fully understand the rationality and limitations of classified recruitment and establish the concept of scientific decision-making in a new scheme of college entrance examination. There is rationality for the implementation of the classified recruitment, but it should not be a “one-size-fits-all” approach. Classified recruitment has a significant positive impact on students’ college satisfaction in “Project 211″ colleges but has a negative impact on the nonacademic satisfaction of students in non“Project 211″ colleges. Therefore, the needs of students at different levels of colleges should be fully recognized when administrative departments of education and universities formulate admissions policies. At the same time, in the new scheme of college entrance examination, there is flexibility and diversity in rules for voluntary admissions, but the scientific nature of policy formulation must also be taken into account. Both large-span and small-span adjustments have a significant negative impact on academic satisfaction, but factors such as institution level and division of liberal arts and sciences will further have different effects on admission results. Therefore, adjustment and admission should further depend on factors of disciplines, colleges, etc.

However, there are deficiencies in this study, which need further improvement and perfection in future research. First, due to sample limitations, that is, research objects are mainly students from colleges in Beijing, the extensibility of research conclusions may need further consideration. Second, the influencing mechanism of whether students are admitted by the first choice on students’ college satisfaction needs to be further explored by subsequent quantitative research or qualitative interviews. Third, it should be noted that since students from “project 211″ colleges account for a large proportion in the sample, the phenomenon reflected in this paper and the law revealed may be mainly the growth process of students in key universities, and its extensibility remains to be discussed.

## Data availability statement

The original contributions presented in the study are included in the article/Supplementary materials, further inquiries can be directed to the corresponding author.

## Ethics statement

The studies involving human participants were reviewed and approved by The Ethics Committee of Tianjin University. The patients/participants provided their written informed consent to participate in this study.

## Author contributions

YH and XL designed the study; YH, MH, HW, ZC, and XL wrote the manuscript and managed the literature analyses. All authors contributed to the article and approved the submitted version.

## Conflict of interest

The authors declare that the research was conducted in the absence of any commercial or financial relationships that could be construed as a potential conflict of interest.

## Publisher’s note

All claims expressed in this article are solely those of the authors and do not necessarily represent those of their affiliated organizations, or those of the publisher, the editors and the reviewers. Any product that may be evaluated in this article, or claim that may be made by its manufacturer, is not guaranteed or endorsed by the publisher.
